# Tunable optical matter: electrostatic repulsion modulates near- and far-field gold nanoparticle arrangements

**DOI:** 10.1039/d5na00926j

**Published:** 2025-12-30

**Authors:** Jim Jui-Kai Chen, Jorge Olmos-Trigo, Boris Louis, Chih-Hao Huang, Susana Rocha, Hiroshi Masuhara, Johan Hofkens, Rafael Delgado-Buscalioni, Roger Bresolí-Obach, Manuel I. Marqués, Marc Mélendez

**Affiliations:** a Department of Chemistry, Katholieke Universiteit Leuven Leuven 3000 Belgium; b Departamento de Física, Universidad de la Laguna San Cristóbal de la Laguna Spain; c Department of Applied Chemistry, National Yang Ming Chiao Tung University Hsinchu Taiwan; d Max Planck Institute for Polymer Research Mainz 55128 Germany; e Departamento de Física Teorica de la Materia Condensada, Condensed Matter Physics Center (IFIMAC), Universidad Autónoma de Madrid Madrid 28049 Spain marc.melendez@uam.es; f AppLightChem, Institut Químic de Sarrià, Universitat Ramon Llull Barcelona 08017 Spain roger.bresoli@iqs.url.edu; g Departamento de Física de Materiales, Condensed Matter Physics Center (IFIMAC), Instituto Nicolás Cabrera (INC), Universidad Autónoma de Madrid Madrid 28049 Spain manuel.marques@uam.es

## Abstract

The dynamics and equilibrium configurations of immersed optically-bound particles are complex phenomena involving several physical mechanisms such as optical forces, electrostatic interactions, and fluid dynamics. In this work, we unravel, using experiments and numerical simulations, the key role played by short-range electrostatic forces. The repulsive interaction among gold nanoparticles is adjusted by changing the salt concentration. When the electrostatic interaction is reduced, near-field optical binding with particles oriented along the polarization direction is promoted, while, for low values of the salt concentration, inter-particle repulsion induces far-field (FF) optical binding configurations oriented perpendicular to the polarization. The importance of electrostatic force is confirmed by a theoretical model in which the repulsive effect is explicitly tuned. The numerical results reproduce the measured particle configurations and highlight the dominant role of electrostatic interactions, particularly in FF optical binding configurations.

## Abstract 2

Experiments and simulations reveal that salt-dependent electrostatic repulsion modulates near- and far-field optical binding of gold nanoparticles, with a simple model reproducing observed configurations and highlighting electrostatics' key role.

## Graphical abstract

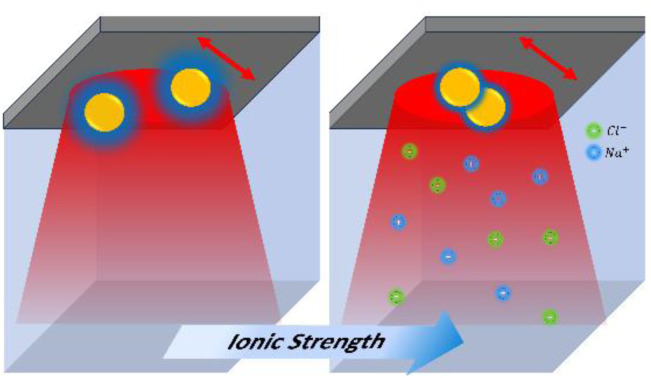

## 1 Introduction

Since Ashkin and colleagues first demonstrated optical trapping of dielectric particles in 1986,^1^ this technique has found widespread applications in manipulating micro- and nanoscale objects with high spatiotemporal precision.^2,3^ Countless advancements have been achieved across various domains, including fundamental research, materials science, and biological studies.^4–7^ However, trapping multiple particles in solution is still a challenge for many different types of materials and particle sizes, as the optical gradient force needs to overcome the scattering force. In contrast, a completely different picture is observed when trapping at interfaces, as both optical forces cooperatively contribute to the particle trapping.^8^ Indeed, optical trapping at solution interfaces enables multiple micro-/nano-scale objects (*e.g.*, dielectric objects, proteins, *etc.*) to form assemblies,^9,10^ which can expand far beyond the irradiated area through the propagation of the optical forces along the assembly as well as coupling with other “non-optical” forces such as capillary or electrostatic forces.^11,12^ These configurations, known as optical matter, are a unique type of non-equilibrium (active) self-assembled structures that remain cohesive only in the presence of an optical field.^13^ The properties of the final material depend on the interactions among the individual components and their specific characteristics (*e.g.*, size, shape, material, type).^14,15^ Therefore, understanding these interactions is crucial for their rational design.

**Fig. 1:**
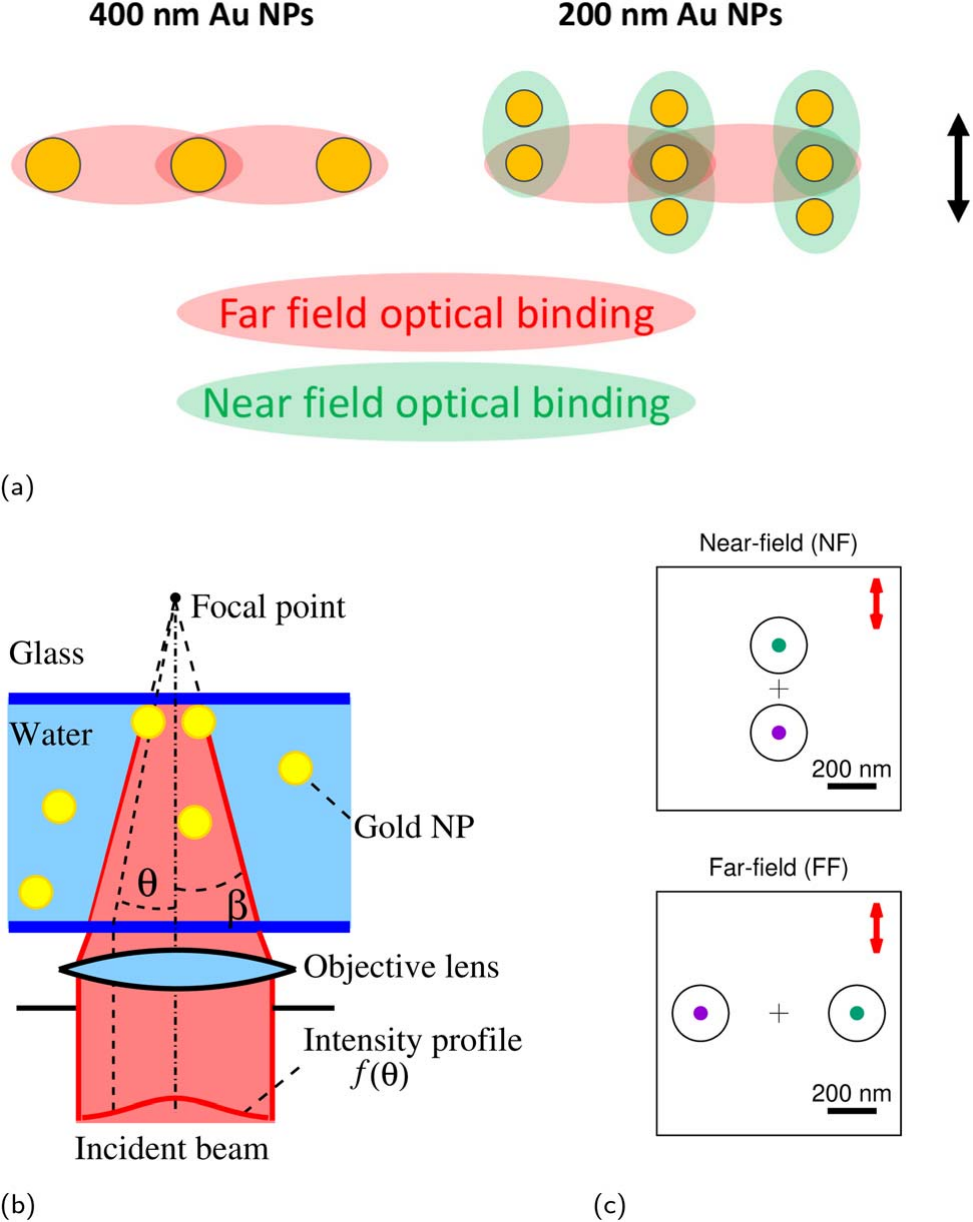
(a) Schematic representation of the experimentally observed configurations for trapped Au NPs (400 nm and 200 nm in diameter), that display a combination of NF and FF binding lengths. The arrow indicates the direction of polarization. The optical binding sketches were drawn based on the configurations reported in ref. 19, 22, 32 and 33. (b) A simplified diagram of an optical trap, displaying a laser beam with an amplitude profile *f*(*θ*) focused to a diffraction-limited spot near a water–glass interface. The numerical aperture equals NA = *n *sin(*β*) and the wavelength *λ* is chosen to trap the suspended Au NPs of polarizability *α*_*j*_. (c) NF and FF configurations for two trapped 230-nm-diameter Au NPs in a plane perpendicular to the incident beam directly below the glass surface. The cross indicates the position of the trap focus and the red arrow denotes the direction of polarization.

More recently, interest has grown in optical trapping of metallic NPs due to their surface plasmon resonance (SPR) properties. The collective oscillation of free electrons on the metal surface significantly enhances the polarizability of these NPs, resulting in amplified optical forces by at least one order of magnitude.^16–18^ Specifically, gold nanoparticles (Au NPs) exhibit a dynamic swarming assembly that extends beyond the focal trapping spot by a few micrometers. These assemblies can be dynamically tuned, reconfigured, and exhibit unique collective behaviors such as flocking, swarming, or propagating waves.^19^ These behaviors arise from interactions within the colloids and with their surrounding medium, making them controllable model systems for developing intelligent swarming nanorobots.^20,21^ Initially, within the focal spot, periodic structures reminiscent of Yagi–Uda antennas assemble perpendicular to the linear laser polarization (FF optical binding), underscoring the pivotal role of the dipole scattering mode of Au NPs in this phenomenon (see Fig. 1a).^22^ These structures find stability through an interparticle radiation force known as optical binding, arising from light scattering interactions between adjacent particles.^13,23^ Ideal optical binding conditions possess some special characteristics such as quantized interparticle distances equal to a multiple of the effective incident trapping wavelength in the medium (*λ*_trap_/*n*_solvent_), and NPs moving cooperatively like a single cohesive body.

However, for smaller-sized particles, another type of optical binding configuration, in which the NPs are aligned parallel to the laser polarization direction and based on near-field interactions, appears and competes with the aforementioned FF optical binding configuration^24,25^ (see Fig. 1a and c). According to previous studies, the preferred optical binding geometry of metallic NPs depends strongly on particle size. Particles larger than roughly 300 nm typically arrange in FF-dominated patterns, whereas those smaller than about 150 nm are governed mainly by NF interactions.^22,26^ In the intermediate range, the configuration attained after trapping can vary with the particles' approach trajectories, similar to collision pathways in molecular systems.^25^ For instance, 200 nm Au NPs can assemble into a Yagi–Uda-type pattern that reflects the coexistence of NF and FF coupling (Fig. 1a).^19^ We can distinguish between FF and NF cases not only by interparticle distance, which arises from optical binding due to the coupling of multipolar plasmon oscillations, but also by considering the potential contribution of van der Waals interactions caused by spontaneous dipole oscillations.^26,27^ Moreover, under some experimental conditions (*e.g.*, substrate,^28^ heating,^8,29^ surfactant,^30^ dipole and quadrupole contribution,^31^ relative position of the laser focus with respect to the interface,^32^ strong electrostatic interaction^26^) the behavior of optically-bound objects deviates from ideality. Previous work has revealed that the configurations adopted by trapped hybrid metallic–dielectric NPs depend on heating of the surrounding water and, especially, on details of their electrostatic interactions. Disregarding these effects may lead to incorrect predictions.^33^ Thus, a more complex interplay of forces in optical systems needs to be developed to describe the coupling of optical binding with other non-optical forces.^34^

In this work, we integrate experimental results and theoretical calculations to develop a generalized numerical model with a low computational cost that describes optical binding between multiple particles by treating them as electric dipoles within a tightly focused Gaussian optical field. We further show that the optical forces predicted by the dipole approximation are in good agreement with those calculated using the full Maxwell stress tensor. This approach is particularly relevant when additional forces beyond optical trapping and binding are significant, as their effects can be systematically included in the framework. Specifically, we study the effect of varying the ionic strength (*i.e.* the electrostatic repulsion) of the suspensions on the dynamics of small optically-bound Au NPs for both NF and FF optical binding configurations. While we acknowledge that electrostatic interactions and their modulation by ionic strength are well-established in colloidal systems and influence optical trapping efficiency and mechanisms,^12,26,34,35^ we show for the first time that this influence goes far beyond a simple tuning of trap stiffness, as it enables an effective selection among different equilibrium configurations in optically bonded matter. Moreover, while we have currently focused on optical binding, gradient, and scattering as optical forces, and electrostatic and hydrodynamic interactions as non-optical forces, the model can be readily extended to incorporate additional forces. Hence, its applicability could be extended to other optically active self-assembling systems that respond to various physical and/or chemical fields. This flexibility opens the door to applying our model to multi-body systems involving a significant number of particles (>10), which constitute the primary components of optical matter.

## 2 Results and discussion

### 2.1 Theoretical framework for modeling optical binding *via* the dipole approximation

This section outlines the algorithm used to carry out the optical trapping simulations. In a nutshell, the focused laser beam is represented as a linear combination of plane waves and the resulting field is calculated as the sum of the incident beam and multiple scattering due to the presence of dipolar particles in the field. Rather than assuming that the field as arises solely from pairwise interactions, we explicitly account for multiple reflections. From this optical field, we calculate the optical force acting on each particle and sum it to the other interactions (mechanical and electrostatic). The particle motion is then simulated using a Brownian Dynamics integration scheme that includes hydrodynamic interactions.

The theoretical model is designed to replicate the experimental conditions. Specifically, a laser beam of wavelength *λ* is focused to a diffraction-limited spot, and traps spherical Au NPs close to a water–glass interface (Fig. 1b). An accurate picture of the dynamic behavior of the particles requires, first, a detailed model of the incident beam. We represent the primary electric field (**E**_0_) due to the laser with a Debye–Wolf decomposition,^36,37^ as a discrete sum of plane waves.1
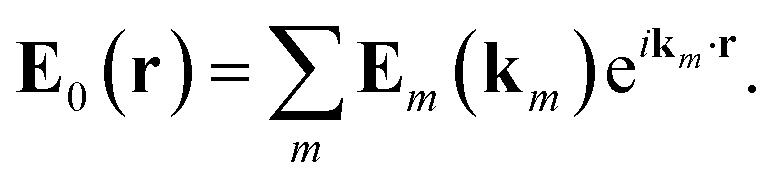


We assign a direction to each wave vector **k**_*m*_ by means of the polar angles *θ*_*m*_ and *ϕ*_*m*_, contained in the solid angle determined by the numerical aperture (NA) of the objective,2
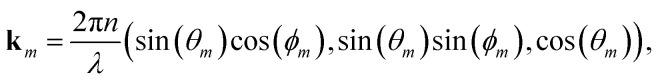
with *n* the refraction index of water 
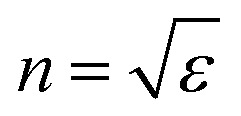
, and *ε* the dielectric constant of the surrounding water medium. Instead of dividing up the solid angle by placing the polar angles into equal intervals, which leads to a greater density of wave vectors close to the focus, we generated a more uniformly distributed set of directions with a Fibonacci lattice.^38^ The simulations presented below described the field with 250 different wave vectors. This number was small enough to allow for a fast calculation of the optical field and forces, but it still provided accurate values of the fields (the error was estimated by comparing the optical field calculated with 250, 1000 and 2000 wave vectors).

For each **k**_*m*_, the complex-valued amplitude **E**_*m*_ must take into account both the effect of the focusing and the intensity of the incident light, which displayed a bell-shaped curve with its maximum at the center of the objective lens.^36,37^3
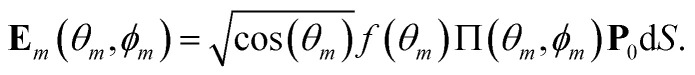


The function *f*(*θ*_*m*_) represents the beam amplitude profile, modeled as a Gaussian function, 
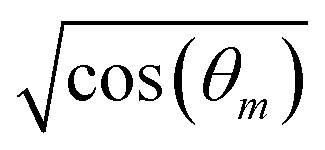
 is the apodization factor, d*S* is the solid angle element, and **P**_0_ determines the polarization (for example, **P**_0_ = (0, 1, 0) produces vertically polarized light, while **P**_0_ = (1, *i*, 0) provides circular polarization). The following rotation matrix imposes the condition of orthogonality.4
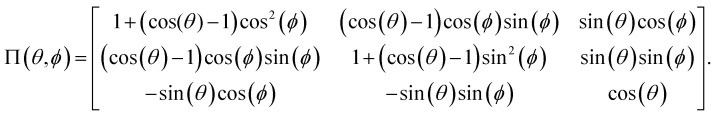


At this level, we disregarded reflection at the water–glass interface, as it contributes less than 1% to the total field.^22^ With this simplification, the total field consists of both the incident beam and the light scattered by the Au NPs. Numerically solving the problem requires evaluating the electromagnetic field in the region surrounding the immersed particles, typically using a mesh with many nodes per sphere.

Discretizing the differential Maxwell equations and solving the resulting system leads to *O*(*M*^3^) computations, where *M* stands for the number of mesh points. In a system with *N* spheres, *N* ≪ *M*, we can drastically reduce the computational cost to *O*(*N*^3^) by approximating each sphere as a point electric dipole and describing the scattered field in terms of a Green function propagator. Similarly, we solve the hydrodynamic interactions with a method based on hydrodynamic Green functions, as explained later on.

We confirmed, by means of Mie scattering calculations, that we can neglect higher order electric and all magnetic contributions to the scattering (see Fig. S1 in SI).^39^ Let *G*(**r**, **r**′) represent the green function propagator for scattered light at point **r** due to a dipole at **r**′.^40^5
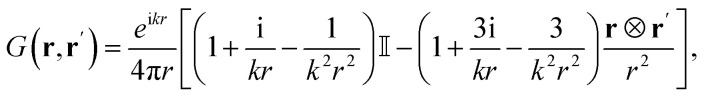
where *r* = ‖**r**′ − **r**‖ and *k* = 2π*n*/*λ*. If we represent the position of particle number *i* with **r**_*i*_, then the total electric field at the position of particle *i* equals^40^6
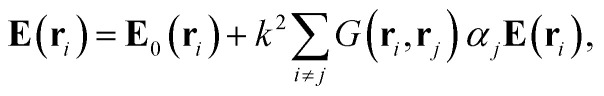
where *α*_*j*_ stands for the polarizability of particle number *j*. Eqn (6) takes the multi-body nature of the scattering process into account explicitly. The total field appears inside the sum on the right, that is to say, particles also scatter light that was scattered by other particles. This makes the equation for the total field implicit. Solving it analytically involves a matrix inversion, a costly operation in simulations, as it would have to be carried out at every time step. Therefore, we found it more convenient to solve the equation for the total field iteratively, by approximating the field inside the sum on the right initially with **E**_0_(**r**_*j*_), calculating the total field, then inserting the result into the sum on the right, and repeating the operation until convergence.

Having determined the total field at the position of every dipolar particle, we must next calculate the optical force exerted on it, which equals^41^7
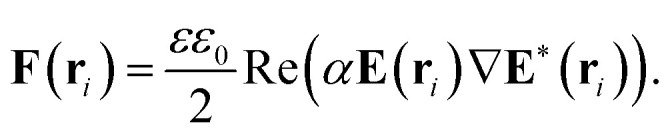


The gradient of the incident field is easily calculated for **E**_0_, but for the total field we need the expressions for **E** and ∇**E** at the positions **r**_*i*_, which are calculated taking into account that the gradient operator in the sum below acts only on the propagator because it involves the derivatives in terms of the **r**_*i*_ but not the **r**_*j*_ coordinates.8
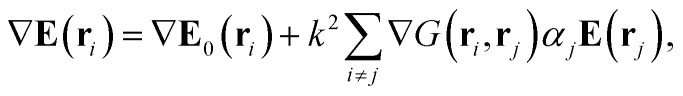


We made sure that eqn (7) reasonably approximated the more exact value determined by solving the scattering fields using finite elements and computing the force by means of the Maxwell stress tensor (see Fig. S2 and S3 in SI). Fig. S3 compares our dipolar approximation for the force in a three-particle configuration with results from the Maxwell stress tensor method, and also includes a linear superposition of pairwise interactions. While the pairwise approach deviates significantly, our multi-body calculation closely matches the Maxwell stress tensor, with equilibrium differences smaller than thermal fluctuation displacements. When compared to the evaluation of the force on two particles by the latter method, we found that our algorithm provides a speedup of approximately six orders of magnitude.

We model steric interactions among spheres and between each sphere and the glass surface with the repulsive Weeks–Chandler–Anderson (WCA) potential^42^9
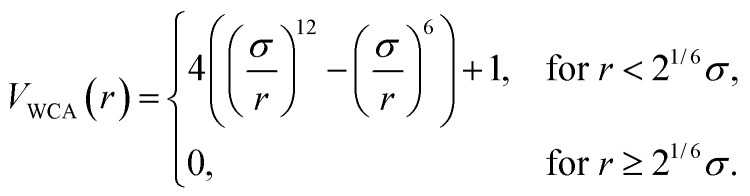
where *r* = ‖**r**_*j*_ − **r**_*i*_‖. Because the Au NPs acquire a negative charge in suspension, they feel a strong repulsive force when they approach each other closely. We choose the *σ* parameter above equal to 1.5 times the diameter of the spheres to account for the repulsion between centers due to the gold core plus the additional electrostatic double layer. Here, we use *σ* only in the calculation of the WCA force. The description of the optical fields and forces relies on the experimental size of the NPs.

Previous work has argued that the details of the electrostatic interaction influence the configurations and dynamics of the optically trapped particles.^33^ As shown below, because the intensity of these forces can be tuned experimentally, they can be used to alter the trapped particle configurations. A more detailed model of the electrostatic interactions, based on Gouy–Chapman theory, states that the potential for two particles of radius *R* equals,^43^10
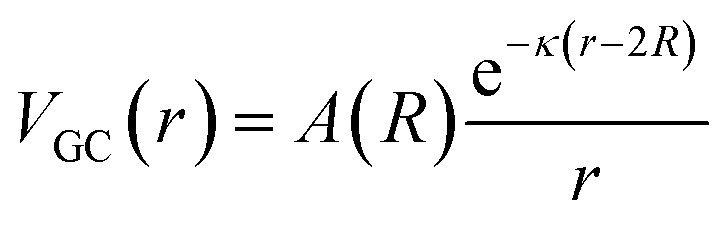
where *A* is a function of the particles' radii,11
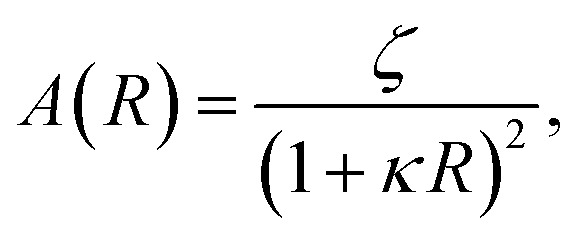
and *ζ* determines the intensity of the potential. We set *ζ* according to the measured value of the zeta potential in pure water (−30 mV, see Fig. S4 in SI). The *κ* parameter in eqn (10) represents the inverse of the Debye length (*λ*_D_), which equals about one micrometer in pure water, but which we can easily decrease by dissolving an ionic salt (*e.g.* NaCl) into the medium. For a 1 : 1 electrolyte, the Debye length in nanometers changes with the molar concentration *M*_e_ according to^44^12
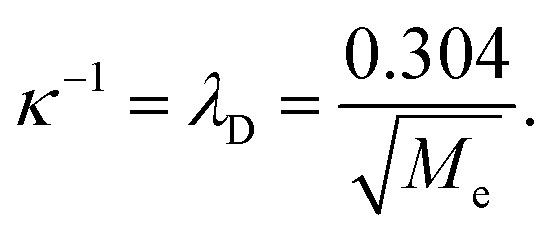


For pure water, we just used *κ*^−1^ = *λ*_D_ = 1000 nm. In the work presented here, simulations of two trapped particles included the more rigorous *V*_GC_ potential.

To describe the motion of the system, we rely on Brownian Dynamics^45^ (overdamped Langevin equations of motion) with hydrodynamic interactions, as represented in the following Itō stochastic differential equation,^46–48^13
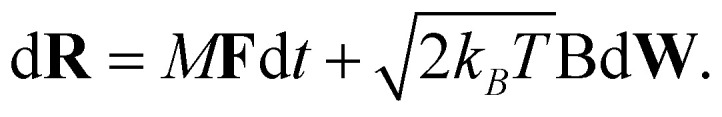


The mobility matrix *M* stands for the Rotne–Prager–Yamakawa tensor,^49,50^ d**W** for the Wiener process, and B is chosen so that BB^T^ = *M* to ensure the fluctuation–dissipation condition is met.^45,51^ In addition, we impose slip boundary conditions on the velocity field at the water–glass interface by means of the method of images. The force 
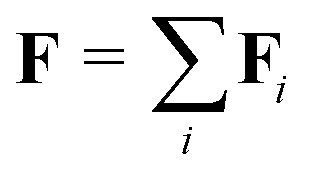
 equals the sum of forces due to all interactions other than hydrodynamics. Here, we incorporated optical, mechanical and electrostatic forces, but the scheme allows for additional effects, like forces derived from potentials (such as gravity, see Fig. S5), magnetic forces, or responses to spatial gradients (such as temperature or chemical concentrations).

To integrate the equations of motion, our simulations rely on a second-order-accurate algorithm^52^ (see SI for more details).

### 2.2 Experimental validation of near- and far-field optical binding

We began by verifying that our model correctly represents the behavior of a small group of Au spheres (230 nm in diameter) trapped in water *n*_H_2_O_ = 1.334 with a *λ* = 1064 nm wavelength laser focused to a spot 1 µm above the water–glass interface (numerical aperture NA = 0.90). Simulations of a single trapped particle were used to tune the intensity of the incident beam, by matching the standard deviation of the particle position histogram to the experimental data (Fig. S6 in SI).

**Fig. 2:**
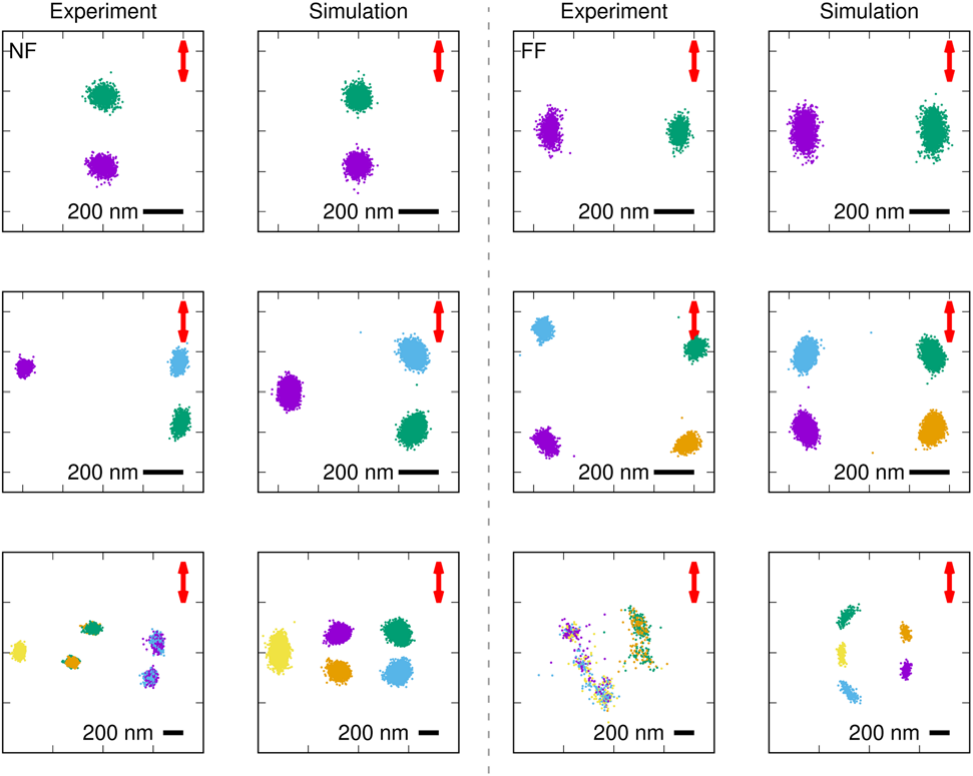
Single particle tracking data for 2–5 trapped Au NPs (230 nm in diameter) as observed in experiments, and corresponding simulations (wavelength *λ* = 1064 nm, numerical aperture NA = 0.90, refraction index *n*_H_2_O_=1.334). Different colors correspond to different particles. The scale bar marks 200 nm and the red arrows indicate the direction of the trapping laser polarization.

Fig. 2 shows the experimental particle tracking data for 2–5 trapped particles systems alongside simulations with numerical parameters chosen to represent equivalent conditions. For the cases with 3 and 5 particles, we observe two configurations: the one shown in Fig. 2 and its mirror image with respect to the vertical axis. The top row displays two different stable configurations for two trapped particles, referred to elsewhere as NF and FF configurations.^23–25^ Experimentally, the FF optical bond is more likely to occur than NF optical bond (55% *vs.* 45%, respectively, Fig. S7). Interestingly, once a particular configuration is established, it does not switch to the other unless the trapping laser is turned off and then back on, indicating a large activation energy barrier between the two configurations. The simulations also exhibit the same kind of stability.

The three- and four-particle configurations on the middle row appear to arise from a combination of NF and FF optical bonds, similar to the Yagi–Uda antenna configurations observed in the many-particle configurations (Fig. 1a). Simulations of five particles predict larger activation energy barriers for rearrangement than observed in experiments, where particles often swap positions. In fact, the arrangement on the bottom right, while stable for the simulated trap, was only occasionally observed experimentally (for periods of about one second) during stochastic particle rearrangement. Similarly, the stable configuration on the bottom left corresponds to a particular experiment. In others, the particles constantly shifted between the two arrangement shown and its mirror image.

What explains the difference between numerical and experimental dynamics? It is tempting to attribute these discrepancies to uncertainties in the exact parameter values. For example, measuring the height of the laser focus accurately is challenging, as the axial resolution of a widefield microscope is no better than 600 nm, and the value chosen here could therefore be off by a few hundred nanometers.^32^ Similarly, we expect the irradiated particles to heat up the water around them, which leads to an increase in the magnitude of thermal fluctuations and induces a thermal gradient in the medium,^53,54^ but accurately estimating the increase in temperature at the working condition is complex and requires its own investigation. Nevertheless, changing the height by a micron or the temperature by about twenty degrees celsius does not appear to have much of an effect on the types of configurations observed. The same is true for hydrodynamic interactions: disregarding them does not introduce qualitative changes (see SI, Fig. S8).

It should be noted that, if scattering forces are neglected, only a single particle can be trapped at the focal center, and none of the structures shown in Fig. 2 appear near the interface (see SI, Fig. S9).

To modulate the arrangement of the structures, we exploit electrostatic interactions as a tunable parameter within the optical binding potential. Through the controlled addition of electrolytes, the electrostatic repulsion between NPs is finely tuned. In combination with the dominant optical forces, this modulation shifts the equilibrium positions, providing opportunity for the NPs to rearrange from one configuration to the other.

### 2.3 Governing role of electrostatic repulsion in near- and far-field optical binding configurations

To unravel the impact of the electrostatic force on the optical binding force, we studied the motion of two optically trapped Au NPs in several suspensions with increasing salt concentration (*i.e.*, different ionic strengths).

**Fig. 3:**
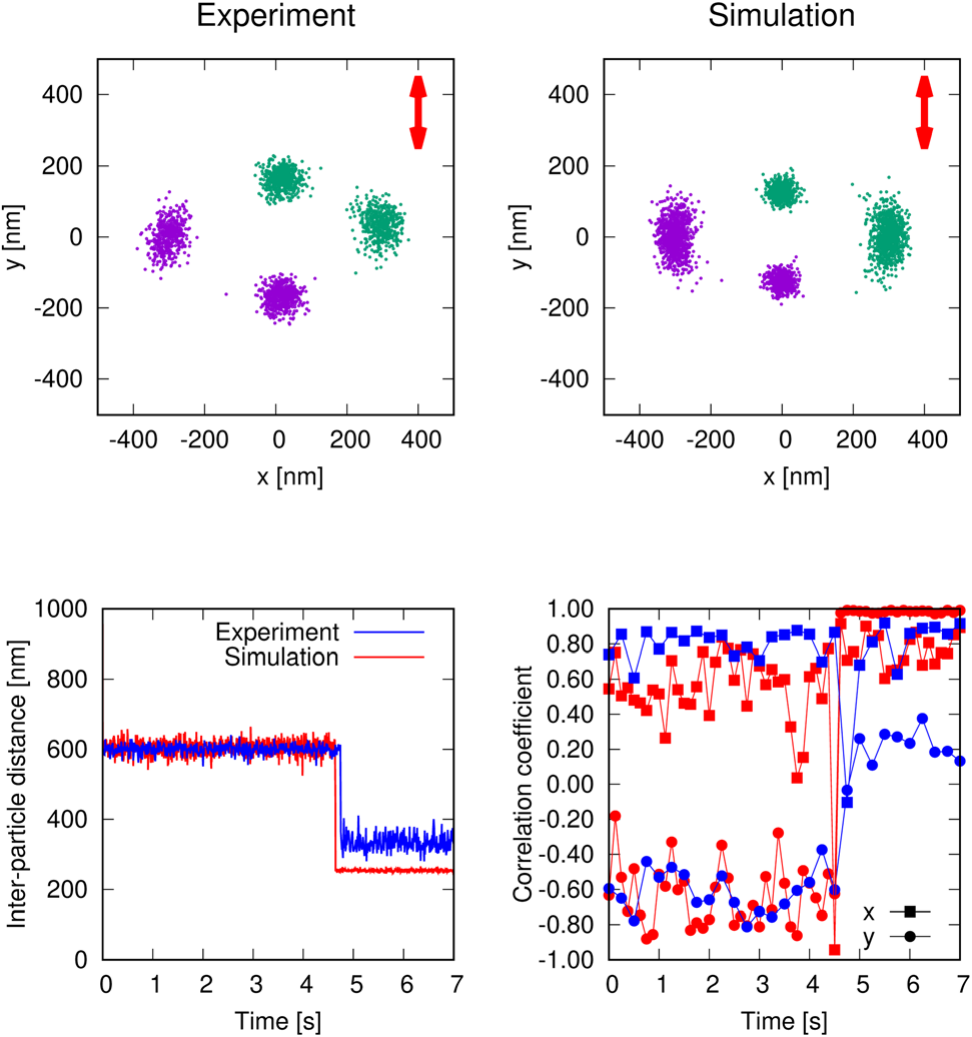
Top panels: Particle tracking data and corresponding simulation for two trapped 230-nm-diameter Au NPs in a 750 µM NaCl solution, switching from the FF to the NF configuration. Bottom left: Inter-particle distance *versus* time. For ease of comparison we chose an experiment–simulation pair with nearly the same transition time (approximately 4.5 s). Bottom right: Pearson correlation coefficient between the coordinates of the particles calculated every 25 frames (0.25 s) *versus* time. Experimental data are shown in blue and simulations in red.

As mentioned before, in the absence of salt, the two trapped particles remain either in the NF or the FF configuration with the latter occurring in 55% of the trials. In simulation, we set the intensity of the Gouy–Chapman electrostatic interaction using the measured ζ-potential and found that the interparticle distance for both the NF (350 nm) and FF (650 nm) configurations agreed with experiments. Grouping the frames into sets of 25 (equivalent to 0.25 s), we measured correlations between two displacements with the Pearson coefficient. The NPs exhibit highly correlated displacements along the direction perpendicular to laser polarization, with a correlation coefficient of 0.84 ± 0.44 (Fig. 3). In the FF configuration, there is also a non-negligible negative correlation in the *y* direction, of −0.43 ± 0.19. Notably, although the negative correlation along the *y* axis was not present for larger particles, it has been observed for silica-shelled particles, where it was attributed to an apparent rotational motion due to a dynamic equilibrium between two conformers.^33^ In this case, the negative correlation indicates the tendency to rotate from the FF to the NF configuration, as the latter exhibits a greater stability when we only consider the optical forces, with the actual relative stability of the FF and NF configurations also influenced by the strength of the electrostatic repulsion. Our simulations qualitatively reproduce the observations for FF case (coefficients of 0.7 and −0.6, respectively) and the orientation and interparticle distance for the NF configuration. We also find some differences in the magnitude of the fluctuations in the NF configuration.

Adding salt (NaCl) to the system causes significant changes due to an increase in the ionic strength. We have observed a considerable increase in the likelihood of the NF configuration due to the thinner double dielectric layer caused by the dissolved ions. In experiments with the laser switched on and off intermittently, allowing particles to diffuse away from the trap for 200 ms, the chance of forming an NF configuration increased to around 70% when 100 µM NaCl was dissolved in the surrounding water, which agrees with numerical results (Fig. S7, in the SI). Simulations also yield interparticle distances similar to those of experiments for both NF and FF configurations (see SI, Fig. S10). Moreover, at a concentration of 750 µM NaCl, the conversion from the FF to NF configuration becomes thermally feasible, as repeatedly observed under this salt condition (Fig. 3). Indeed, the experiments reveal a reversal in the thermodynamically most stable configuration: under high salt concentrations, the NF optical bond becomes the most probable, occurring in approximately 70% of cases. At even higher NaCl concentrations, exceeding 1000 µM, the two-NP arrangement becomes unstable. They irreversibly fuse into a dimer as soon as they approach each other due to the combined attractive effect of the NF optical binding and the optical gradient force. Under these conditions, the electrostatic repulsion is too weak to prevent irreversible NP aggregation.

Numerical results further confirm that variations in salt concentration modulate the relative stability of the NF and FF configurations within the optically dominated trapping potential. The effect of changing ionic strength is modeled by adjusting the Debye length in the Gouy–Chapman potential (10). At a concentration of 750 µM, both experiments and simulations reveal transitions from FF to NF configurations occurring over timescales of a few hundred milliseconds to several seconds (Fig. 3), consistent with electrostatic modulation of the optically defined potential landscape. The tracking data aligns well with simulation positions and the evolution of the interparticle distance reveals the sharp switch between configurations. Our model correctly describes the FF configuration and yields a reasonable prediction for the NF interparticle distance, which it underestimates slightly.

**Fig. 4:**
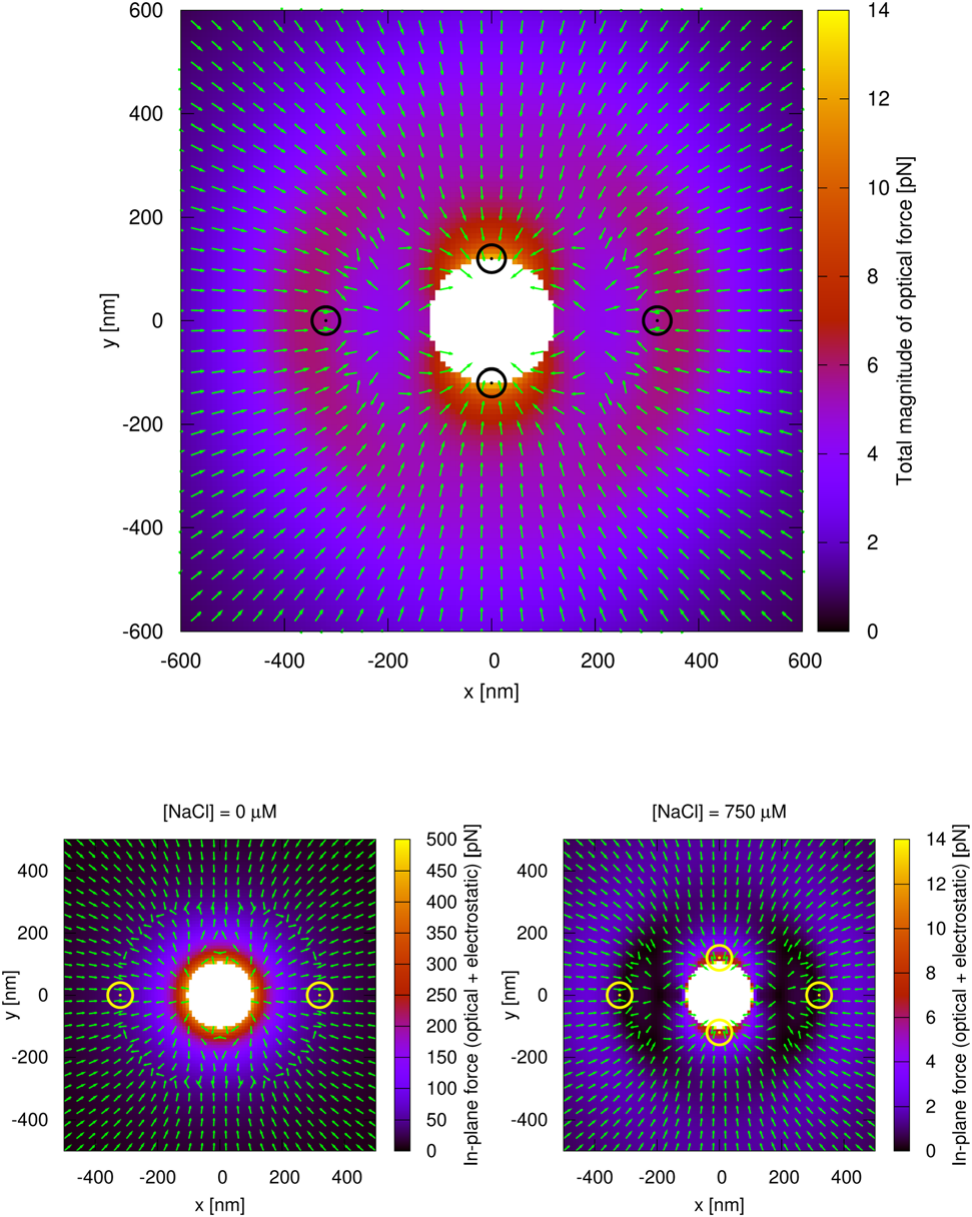
Top: Optical force field on a 230 nm diameter dipolar trapped Au NP created by both the incident beam and the scattering off another identical particle placed symmetrically with respect to the origin of coordinates. The white area represents an inaccessible region, as it would imply particle overlap. The color scale indicates the total magnitude of the optical force on the NP, while the green arrows mark the direction of the component of the force within the *x*, *y* plane. The circles mark points of stable equilibrium. Bottom: Combined force field due to optical forces and electrostatic repulsion for two different salt concentrations. In pure water (left) the repulsion between particles is much stronger, so they tend to move towards the FF configuration. At higher salt concentrations (right), particles can switch from FF to NF due to thermal fluctuations.

Fig. 4 (top panel) displays the pure optical force field for a trapped particle assuming that there is another trapped particle placed symmetrically with respect to the origin of coordinates, revealing the stable NF and FF configurations. In the bottom panel of Fig. 4, the combined force field due to optical forces and electrostatic repulsion is considered for two different salt concentrations. As the electrostatic repulsion between particles decreases with growing salt concentration, the probability of trapping in the NF configuration along the *y* axis increases.

Particle interaction is highly sensitive to distance, as previously reported.^22,24,25^ By graphing the *x* and *y* motion correlation coefficients over 0.25 s intervals (25 frames) against average interparticle distances, we can detect significant differences between these two configurations. In the FF configuration, the correlation coefficient between the *x* coordinates of the NPs exhibits an increase concurrent with the rising interparticle distance, while the *y* correlation coefficient may be situated within the standard deviation error (see SI, Fig. S11). For the NF configuration, both *x* and *y* correlation coefficients show an increase with decreasing interparticle distance, suggesting closely synchronized displacements in the presence of salt. Notably, correlations in the fluctuations of position due to hydrodynamics are more significant in the NF configuration, but entail only a minor contribution to the FF correlations. Simulations also reproduce a change in correlations from 0.6 and −0.6 in the *x* and *y* directions, respectively, for FF configurations (similar to experimental data) to very strong positive correlations (0.8 and almost 1.0) after the transition to NF positions (Fig. 3). After 4.5 s, there is a point with an *x* correlation value well below the other positive values observed, both in simulation and experiment. It corresponds to the calculation of the coefficient over a series of frames in which the particles were transitioning from the FF to the NF arrangement, and so the correlation does not match either the FF or the NF configuration.

The only correlations that show a significant deviation from the experimental data are those related to the *y*-coordinate in the NF configuration. The simulations visibly constrain the interparticle distance more than experiments. The cause of this discrepancy in the elasticity of the NF optical bond is likely a combination of several factors. Firstly, the theoretical model of the optical field might differ slightly from the actual beam achieved in experiments, especially in the direction of polarization (which applies to the NF configuration), as suggested by comparison with direct measurements of the incident field intensity (see SI, Fig. S12). Secondly, as the particles approach closely, short-range interactions not captured by the dipole approximation (such as van der Waals attraction) may become more significant.^43^ A more detailed treatment of these forces would be necessary when analyzing fluctuations and correlations in the NF configuration. Thirdly, the accuracy of the tracking is reduced for particles located at very close distances, when the point spread function of both particles is extensively mixed.^55^ This is especially true for the NF configuration case, introducing noise on the experimental correlation coefficients. Lastly, the dipolar approximation is expected to have reduced accuracy at surface-to-surface separations comparable to or smaller than the particle radius^56^ (see SI, Fig. S2).

Despite the limitations discussed, the model remains a valuable tool for understanding optical binding phenomena. Its predictive capability, combined with the tunability of interparticle interactions, provides key insights into the control of NF and FF configurations of plasmonic NPs, making it broadly applicable to the rational design of complex NP systems. Indeed, the electric dipole approximation enables efficient simulation of many-particle dynamics due to its low computational cost, and despite its simplicity, it produces optical forces that closely match those obtained using the full Maxwell stress tensor or the discrete dipole approximation.

## 3 Conclusion

Both the numerical model and experimental observations demonstrate that short-range electrostatic interactions significantly influence the positions and dynamics of optical binding configurations, making the salt concentration (*i.e.*, ionic strength) a tunable parameter for controlling interparticle binding. At higher salt concentrations, the reduced range of electrostatic repulsion facilitates the formation of NF optical bonds. The numerical results show that our model captures the essential trends and provides a reliable description of particle configurations, particularly in the case of FF optical bonds. Importantly, the electric dipole approximation enables simulations at a lower computational cost, making it feasible to investigate many-particle systems that would otherwise be intractable with more demanding approaches such as the Maxwell stress tensor. Moreover, the model can be readily extended to include additional forces and scaled to larger systems. These features make it a practical and versatile framework for exploring non-equilibrium (active) self-assembly driven by physical and chemical fields, ultimately contributing to the rational design of the next generation of functional NP architectures.

## 4 Materials and methods

### 4.1 Sample preparation

A Au NPs suspension (228 ± 20 nm, as determined by a field emission SEM, Quanta FEG250), purchased from BBI solutions, was diluted by different ratios of miliQ water and 1 mM NaCl solution to obtain the desired salt concentrations, 0 µM, 100 µM, 500 µM and 750 µM. For optimal imaging conditions, a particle density (7.3 × 10^−6^ particles per µm^3^) was specifically selected. The ζ-potential of the Au NPs suspension was checked by ZETASIZER NANO ZSP (Malvern Instrument) with a cuvette (DTS1070). To prevent aggregation, the suspension was sonicated for 5 minutes before utilization. Subsequently, 10 µL of the colloidal suspension was sandwiched between two glass cover slides (no. 1) with an imaging spacer (Electron Microscopy Sciences, 20 mm dia × 0.12 mm depth). To avoid NP sticking to the cover slides, the cover slides were treated with a UV ozonator (Ultra-Violet Product, PR-100) for 60 minutes.

### 4.2 Optical setup and single-particle tracking

To achieve a focused laser beam suitable for optical trapping, a 1064 nm laser source (Laser Quantum, opus 1064) undergoes expansion through a 4× beam expander, utilizing a pair of lenses (focal lengths: 25 mm, 100 mm), with the aim of completely filling the back aperture of a 60× air-immersion objective lens (Olympus, UPlanFL N, 60×, NA 0.9). Following the focus through the objective lens, the laser power is finely adjusted to 30 mW by manipulating the polarizer positioned in front of the polarization beam splitter. The laser on/off periods are well controlled by utilizing an optical beam shutter (Thorlabs, SH1/M with SC10 Controller). Given the pronounced scattering characteristics of Au NPs, dark field microscopy is adopted for imaging by replacing the standard bright field condenser with an oil immersion dark field condenser lens (Olympus, U-DCW, IX-ADUCD, NA 1.2–1.4). For optimal tracking and signal-to-noise ratio considerations, images are captured with an exposure time of 10 ms, employing a CMOS camera (Hamamatsu, C11440, ORCA flash 4.0). The image processing and the single-particle tracking (SPT) of the Au NPs were performed as previously reported.^22,57,58^ Briefly, multiple Gaussian curves are simultaneously fitted at the initial positions where the particles were detected, and tracking is performed using the Munkres algorithm to minimize the squared distance. The Pearson coefficient (defined as the covariance of two variables divided by the product of their standard deviations) is then calculated based on the resulting tracked positions.

## Author contributions

Jim Jui-Kai Chen: validation, formal analysis, investigation, writing – original draft, writing – review & editing, visualization. Jorge Olmos-Trigo: methodology, investigation, writing – review & editing. Boris Louis: methodology, software, formal analysis, writing – review & editing, supervision. Chih-Hao Huang: investigation, writing – review & editing. Susana Rocha: validation, formal analysis, writing – review & editing, funding acquisition. Hiroshi Masuhara: conceptualitzation, writing – review & editing, project administration, funding acquisition. Johan Hofkens: conceptualitzation, writing – review & editing, project administration, funding acquisition, supervision. Rafael Delgado-Buscalioni: conceptualitzation, software, resources, data curation, writing – review & editing, project administration, funding acquisition. Roger Bresolí-Obach: conceptualitzation, methodology, writing – original draft, writing – review & editing, visualization, supervision, project administration, funding acquisition. Manuel I. Marques: conceptualitzation, methodology, writing – review & editing, visualization, supervision, project administration, funding acquisition. Marc Mélendez: conceptualitzation, methodology, software, validation, formal analysis, writing – original draft, writing – review & editing, visualization.

## Conflicts of interest

There are no conflicts to declare.

## Supplementary Material

NA-008-D5NA00926J-s001

NA-008-D5NA00926J-s002

## Data Availability

The developed code for particle tracking and its subsequent analysis is available at the following link: https://github.com/BorisLouis/goldTracking.gi. Dedicated code for the theoretical model is available in supplementary information (SI). Supplementary information: additional information, including additional details about the Numerical Simulation of Brownian Dynamics with Hydrodynamic Interactions; 12 supporting figures. Fig. S1 shows that scattering and extinction of 230 nm Au NPs at 1064 nm are dominated (>97%) by the electric dipole response, validating dipolar approximations. Fig. S2 compares NF/FF optical forces *versus* interparticle distance, demonstrating consistency between dipolar models and full Maxwell‑stress‑tensor simulations. Fig. S3 highlights many‑body optical interactions by contrasting plane‑wave and focused‑beam forces and showing that pairwise superposition fails to reproduce full scattering‑mediated forces. Fig. S4 provides the measured ζ-potential of the Au NPs, confirming their negative charge. Fig. S5 demonstrates that optical forces exceed gravity, governing particle trajectories near the trap focus. Fig. S6 presents probability distributions of trapped‑particle positions, with simulations matching experimental fluctuations. Fig. S7 quantifies how NaCl concentration affects NF/FF configuration probabilities under intermittent trapping. Fig. S8 shows simulated multi‑particle configurations (3–4 NPs) and confirms that temperature, focal height, and hydrodynamic interactions do not qualitatively alter the resulting optical matter geometries. Fig. S9 demonstrates that including scattering forces could yield optical binding of 5 gold particles in an optical trap, whereas omitting them produces single‑particle trapping only. Fig. S10 correlates interparticle distances with NaCl concentration for both NF and FF arrangements in experiments and simulations. Fig. S11 reports Pearson correlation coefficients of particle motions, revealing the salt effect on configuration‑dependent coordinated fluctuations. Finally, Fig. S12 compares experimental laser‑intensity profile and analytical one used in simulations. See DOI: https://doi.org/10.1039/d5na00926j.
