# Supplementary material for: Tunable optical matter: electrostatic repulsion modulates near- and far-field gold nanoparticle arrangements
Source: Nanoscale Adv. 2025 Dec 30;8(4):1251–9. doi: 10.1039/d5na00926j (PMC12794035; doi:10.1039/d5na00926j)
Supplement: NA-008-D5NA00926J-s001 [file NA-008-D5NA00926J-s001.pdf]

Supplementary Information (SI) for Nanoscale Advances.
This journal is © The Royal Society of Chemistry 2026

Tunable Optical Matter: Electrostatic Repulsion Modulates Near- and Far-Field Gold Nanoparticle Arrangements

(Supporting Information)

Jim Jui-Kai Chen,[†] Jorge Olmos-Trigo,[‡] Boris Louis,[†] Chih-Hao Huang,[¶] Susana Rocha,[†] Hiroshi Masuhara,[¶] Johan Hofkens,[†,§] Rafael Delgado-Buscalioni,[‖] Roger Bresolí-Obach,[*,⊥] Manuel I. Marqués,[*,#] and Marc Mélendez[*,‖]

†*Laboratory for Photochemistry and Spectroscopy, Division for Molecular Imaging and Photonics, Department of Chemistry, Katholieke Universiteit Leuven, Leuven 3000, Belgium.*

‡*Faculty of Optics and Optometry, Universidad Complutense de Madrid, Madrid 28037, Spain.*

¶*Department of Applied Chemistry, National Yang Ming Chiao Tung University, Hsinchu 300093, Taiwan.*

§*Max Planck Institute for Polymer Research, Mainz 55128, Germany.*

‖*Departamento de Física Teorica de la Materia Condensada and Condensed Matter Physics Center (IFIMAC), Universidad Autónoma de Madrid, C. Francisco Tomás y Valiente, 7, 28049 Madrid, Spain.*

⊥*AppLightChem, Institut Químic de Sarrià, Universitat Ramon Llull, Barcelona 08017, Spain.*

#*Departamento de Física de Materiales and Condensed Matter PhysicsCenter (IFIMAC) and Instituto Nicolás Cabrera (INC), Universidad Autónoma de Madrid, 28049, Madrid, Spain*

E-mail: roger.bresoli@iqs.url.edu; manuel.marques@uam.es; marc.melendez@uam.es

Numerical Simulation of Brownian Dynamics with Hydrodynamic Interactions

The dynamic behavior of the nanoscale gold spheres described above is governed by Brownian motion in a low Reynolds number liquid environment. In this regime, particles quickly accelerate to terminal velocities, as friction from the fluid quickly balances the other forces acting on the particles.[1] Over the time scales of interest for the problem of optical trapping considered here, the acceleration to terminal velocity may be considered instantaneous, so Newton's equation of motion transforms into a relation between the forces and the velocities, namely, the velocity of a particle equals the force times the mobility.

If we disregard the effect of thermal fluctuations, then the system freezes as soon as all other forces stop acting on the particles. Therefore, we must also include a stochastic force to recover the correct equilibrium behavior of the particles for the desired temperature. In the absence of all other forces, particles will diffuse following a Brownian walk.

The following Itō stochastic differential equation expresses the differential displacement for the particle positions $\mathbf{R}$.[2–4]

$$d\mathbf{R} = M\mathbf{F}dt + \sqrt{2k_BT}\ Bd\mathbf{W}. \tag{S1}$$

On the right, $M\mathbf{F}$ stands for the mobility tensor times the deterministic forces due to optics, mechanics and electrostatics. The second term in the sum stands for the random Brownian displacement. The $\mathbf{R}$ and $\mathbf{F}$ vectors contain a list of the three components of position and force for each and every particle.

The force vector $\mathbf{F}$ combines all the optical forces described in detail in the main text, in addition to steric interactions, which describe collisions among the spheres and with the glass wall, and also electrostatic interactions due to the negative charge on the surfaces of the immersed particles.

We used the Weeks-Chandler-Andersen (WCA) potential to model steric collisions among spheres and with the glass surface.[5]

$$V_{\mathrm{WCA}}(r) = \begin{cases} 4\left(\left(\frac{\sigma}{r}\right)^{12} - \left(\frac{\sigma}{r}\right)^{6}\right) + 1, & \text{for } r < 2^{1/6}\sigma, \\ 0, & \text{for } r \geq 2^{1/6}\sigma. \end{cases} \tag{S2}$$

This piecewise function vanishes when particles separate, but when they approach too closely it creates a large repulsive force that prevents them from overlapping. The σ parameter equals the sum of the radii of a pair of interacting spheres, and r the distance between the sphere centers. For collisions with the wall, r indicates the distance from the center of the sphere to the wall, and σ equals the particle radius.

To deal with electrostatic repulsion, in simulations with more than two particles, we have added an ad hoc extra shell of excluded volume around the spheres to account for the electric double layer predicted by Gouy-Chapman theory.[6] However, to study the electric repulsion and its dependence on the concentration of dissolved salt, we relied on a more rigorous representation of the forces in the simulations with two spheres, with the potential represented by

$$V_{\mathrm{GC}}(r) = \frac{Z}{(1+\kappa R)^2} \frac{e^{-\kappa(r-2R)}}{r}, \tag{S3}$$

as explained in the main text. The constant κ above depends on the molar concentration M_e of the dissolved 1:1 electrolyte, according to

$$\kappa = \frac{\sqrt{M_e}}{0.304}, \tag{S4}$$

with the result measured in nm^{-1}.

Because of the surrounding liquid medium, the forces acting on a particle do not only cause displacements for that particle. As it moves, it drags the surrounding fluid with it displacing other particles. This applies, of course, to the forces on all particles, making the

calculation of displacements from forces a multi-body problem. We used the Rotne-Prager-Yamakawa mobility tensor to model the hydrodynamic coupling among particles.[7,8] Given a list of force components $\mathbf{F}$, the product $M\mathbf{F}$ returns a list of corresponding displacements. The boundary conditions at the glass wall were achieved by the method of images, that is to say, for each particle we placed a virtual particle symmetrically with respect to the boundary to ensure that the fluid velocity field did not flow through the wall.

The random Brownian motion was created starting with the Wiener process $d\mathbf{W}$, a list of continuous random displacements with zero mean and variance equal to dt. We must choose the magnitude of these random displacements in such a way as to ensure that the fluctuation-dissipation condition is met at equilibrium,[9,10] which requires that $BB^T = M$. In the numerical algorithm, we obtain an appropriate B at each time step by means of Cholesky decomposition.

Combining the deterministic and random displacements, we get a stochastic differential equation. Numerical trajectories are typically determined by dividing time into small discrete steps and using an appropriate integration scheme to determine the paths in small increments, with Euler-Maruyama being one of the simplest and most popular. Here we employ a slight modification of this algorithm which improves its accuracy to second order. The scheme emerges as the over-damped limit of a Langevin dynamics integrator.[11]

The complete algorithm was written from scratch in C with the specific aim of simulating the dynamics of immersed metallic nanoparticles in optical fields.

References

(1) E. M. Purcell, *Life at low Reynolds number*, American Journal of Physics **45**, 3–11 (1977).

(2) Doyle PS, Underhill PT, *Brownian Dynamics Simulations of Polymers and Soft Matter*, In: Yip S, editor, *Handbook of Materials Modeling* [Internet]. Dordrecht:

Springer Netherlands; 2005 [cited 2024 Jan 26]. p. 2619–30. Available from: `http://link.springer.com/10.1007/978-1-4020-3286-8_140`

(3) Delgado-Buscalioni R, Meléndez M, Luis-Hita J, Marqués MI, Sáenz JJ, *Emergence of collective dynamics of gold nanoparticles in an optical vortex lattice*, Physical Review E, **98(6)**, 062614 (2018).

(4) Meléndez M, Alcázar-Cano N, Peláez RP, Sáenz JJ, Delgado-Buscalioni R. *Optofluidic control of the dispersion of nanoscale dumbbells*, Physical Review E. **99(2)** 022603 (2019).

(5) Andersen HC, Weeks JD, Chandler D, *Relationship between the Hard-Sphere Fluid and Fluids with Realistic Repulsive Forces*, Physical Review A, **4(4)**, 1597–607 (1971).

(6) Israelachvili JN, *Intermolecular and surface forces. 3rd ed*, Burlington (Mass.) Academic press (2011).

(7) Rotne J, Prager S, *Variational Treatment of Hydrodynamic Interaction in Polymers*, Journal of Chemical Physics **50(11)**, 4831–7 (1969).

(8) Yamakawa H, *Transport Properties of Polymer Chains in Dilute Solution: Hydrodynamic Interaction*, Journal of Chemical Physics 53(1) 436–43 (1970).

(9) Fixman M, *Simulation of polymer dynamics. I. General theory*, Journal of Chemical Physics, **69(4)**, 1527–37 (1978).

(10) Delong S, Usabiaga FB, Delgado-Buscalioni R, Griffith BE, Donev A, *Brownian dynamics without Green's functions*, Journal Chemical Physics **140(13)**, 134110 (2014).

(11) Leimkuhler B, Matthews C, *Rational Construction of Stochastic Numerical Methods for Molecular Sampling*, Applied Mathematics Research eXpress **1**, 34–56 (2013).

Supporting Figures

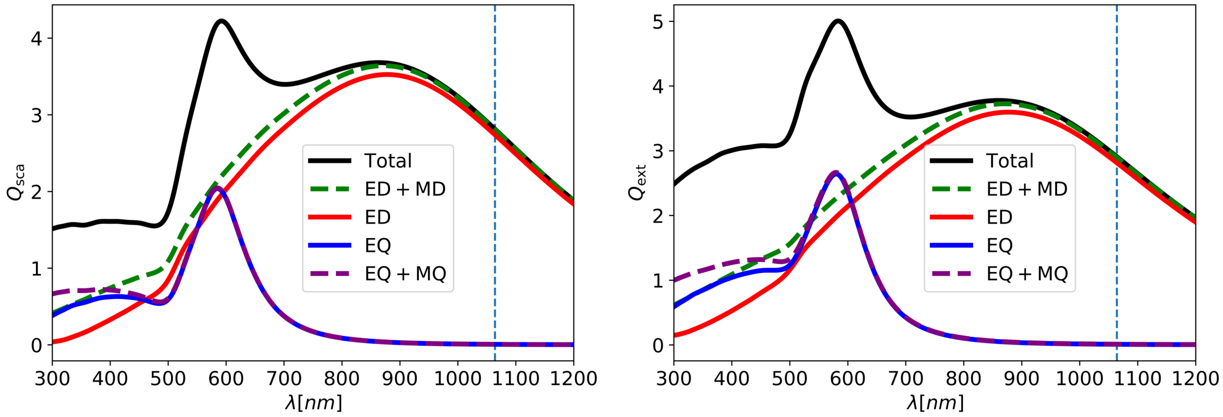

Figure S1: Scattering (*left*) and extinction (*right*) efficiencies spectra for 230-nm-diameter Au NPs. At a wavelength of 1064 nm (dashed line) the dipolar electric contribution accounts for more than 97% of the total cross section.

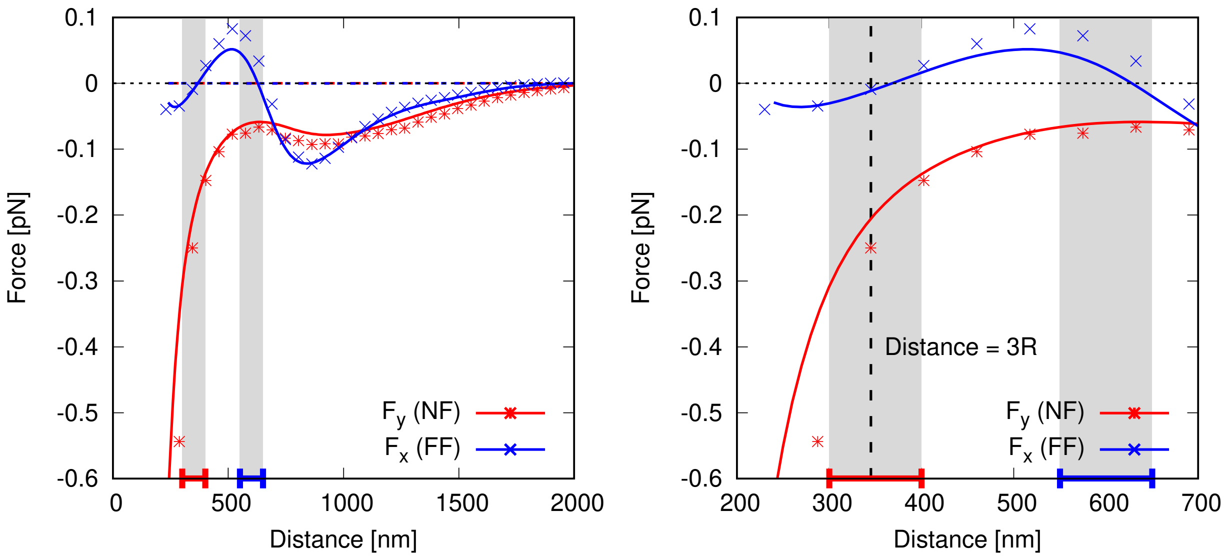

Figure S2: Force on a trapped NP in the NF or FF configuration versus distance between the particle centers. The panel on the right provides a close up of the same data at the distances relevant for experimental NF and FF configurations. Solid red and blue lines present the forces as calculated with the dipolar approximation, while the points were obtained in COMSOL by solving the scattering fields using finite elements and computing the force by means of the Maxwell stress tensor. We have marked the separation of three times the radius between the centers of the spheres as well as the experimental ranges within which we find particles in the NF (red) and FF (blue) configurations.

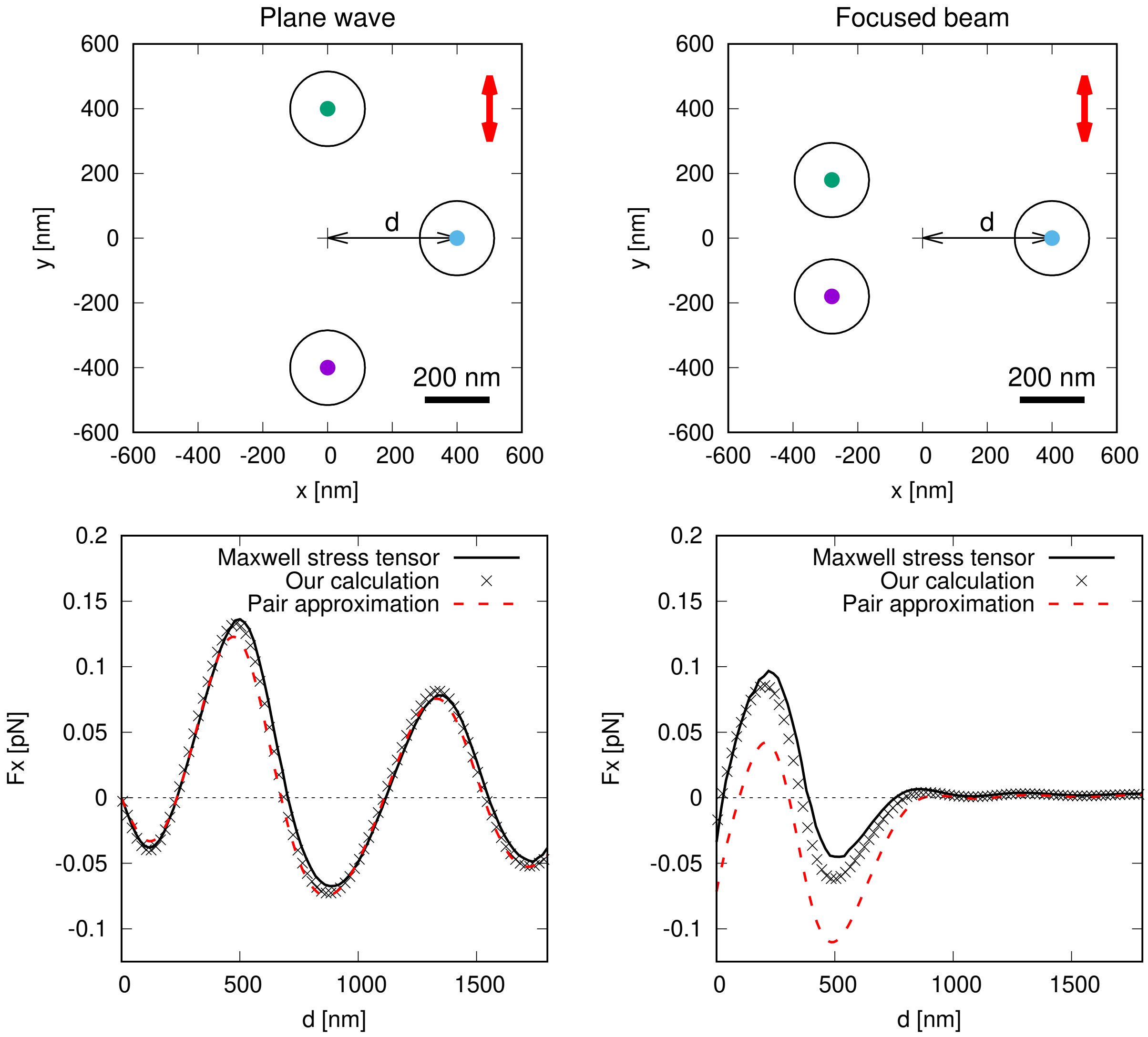

Figure S3: Optical force in the x direction for a 230-nm-diameter Au NP on the x axis due to a beam ($\lambda = 1064$ nm) incident along the z direction with polarisation along the y direction (red arrows) plus the optical field caused by the multiple scattering of light due to the presence of three Au NPs in the field. The panels on the left present results for an incident plane wave, while the ones on the right correspond to a focused Gaussian beam. The lower panels plot the force on the particle on the x axis versus its distance to the origin of coordinates d. We compare the force calculated numerically with the Maxwell stress tensor in COMSOL (solid line) to the dipolar approximation used in the main text (points). The dashed red line presents the force calculated as a linear superposition of pair interactions, which in some cases can differ significantly from the actual forces that arise, proving that simulations require a determination of the many-body scattering field. Note that the differences in the point of equilibrium predicted by the two numerical methods in black lie below the typical displacements observed due to thermal fluctuations.

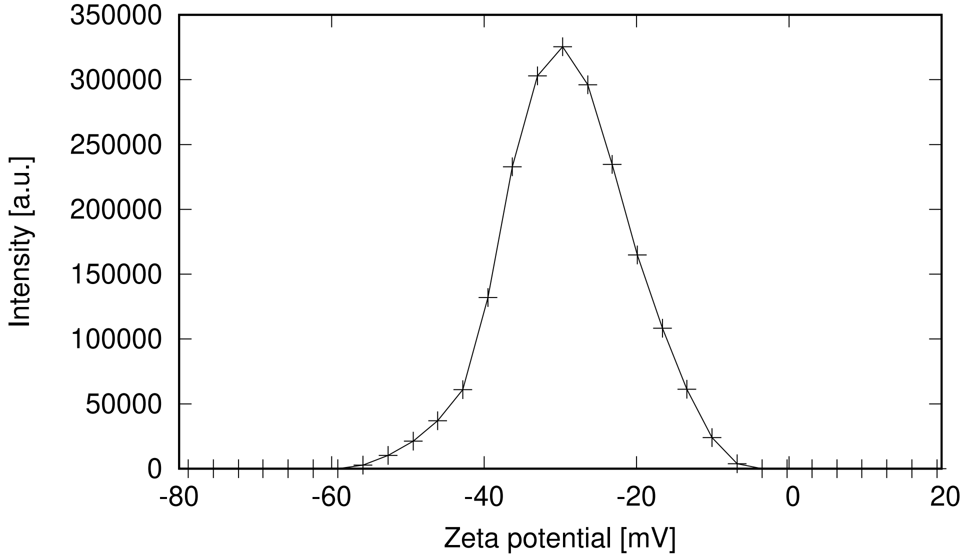

Figure S4: Measured zeta potential for 230 nm diameter gold particles suspended in MiliQ water. The vertical axis measures the scattering intensity of the nanoparticles with a He-Ne 633 nm laser. The peak position on the x axis corresponds to the Zeta potential of the nanoparticles.

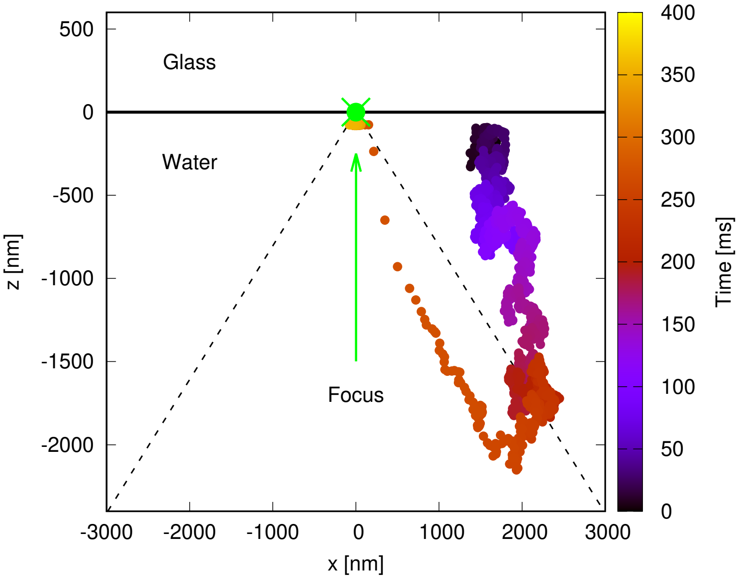

Figure S5: Optical forces overwhelm gravity for trapped particles, so we have disregarded them in the main text. However, weight and buoyancy can be added to describe the trajectory of a particle far from the focus, as shown here. The nanoparticle sinks until it reaches the irradiated cone slightly in front of the xz plane, at which point the optical force becomes dominant and it is propelled towards the focus.

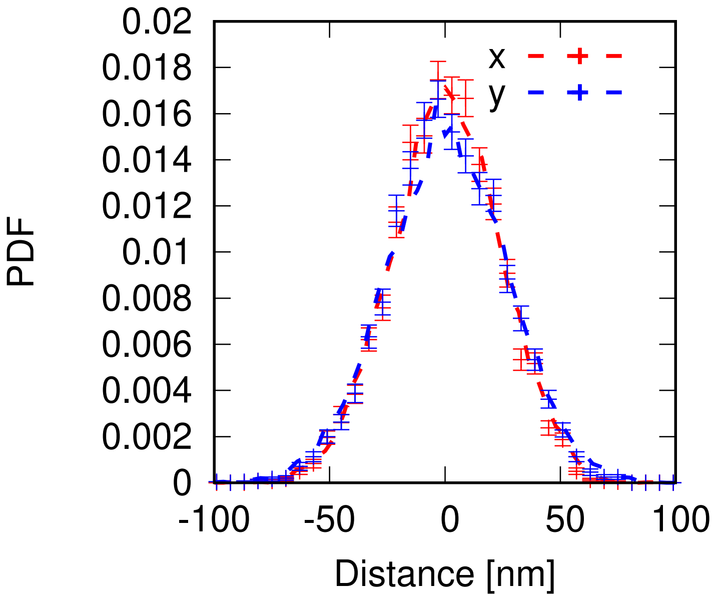

Figure S6: Probability distribution functions (PDF) for the x and y coordinates for a particle trapped in an optical tweezer. The points represent experimental data and the dashed line simulation results.

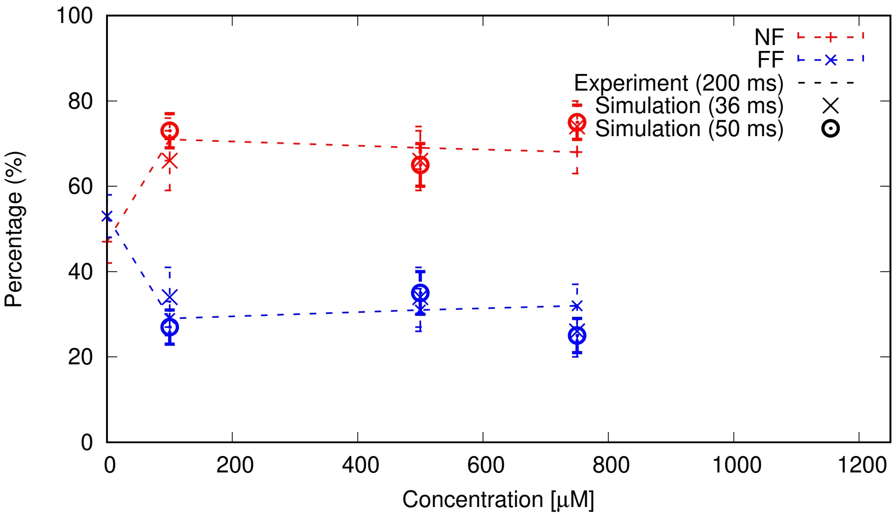

Figure S7: Percentage of two 230 nm diameter Au NPs trapped in the near-field (NF) and far-field (FF) configurations versus NaCl salt concentration when the optical trap was switched ON/OFF intermittently. The key indicates the time between successive trap activations for each set of results. Of note, simulations at 0 μM are not shown because particles diffused away from the trap and escaped permanently after only a few switch ON/OFF cycles.

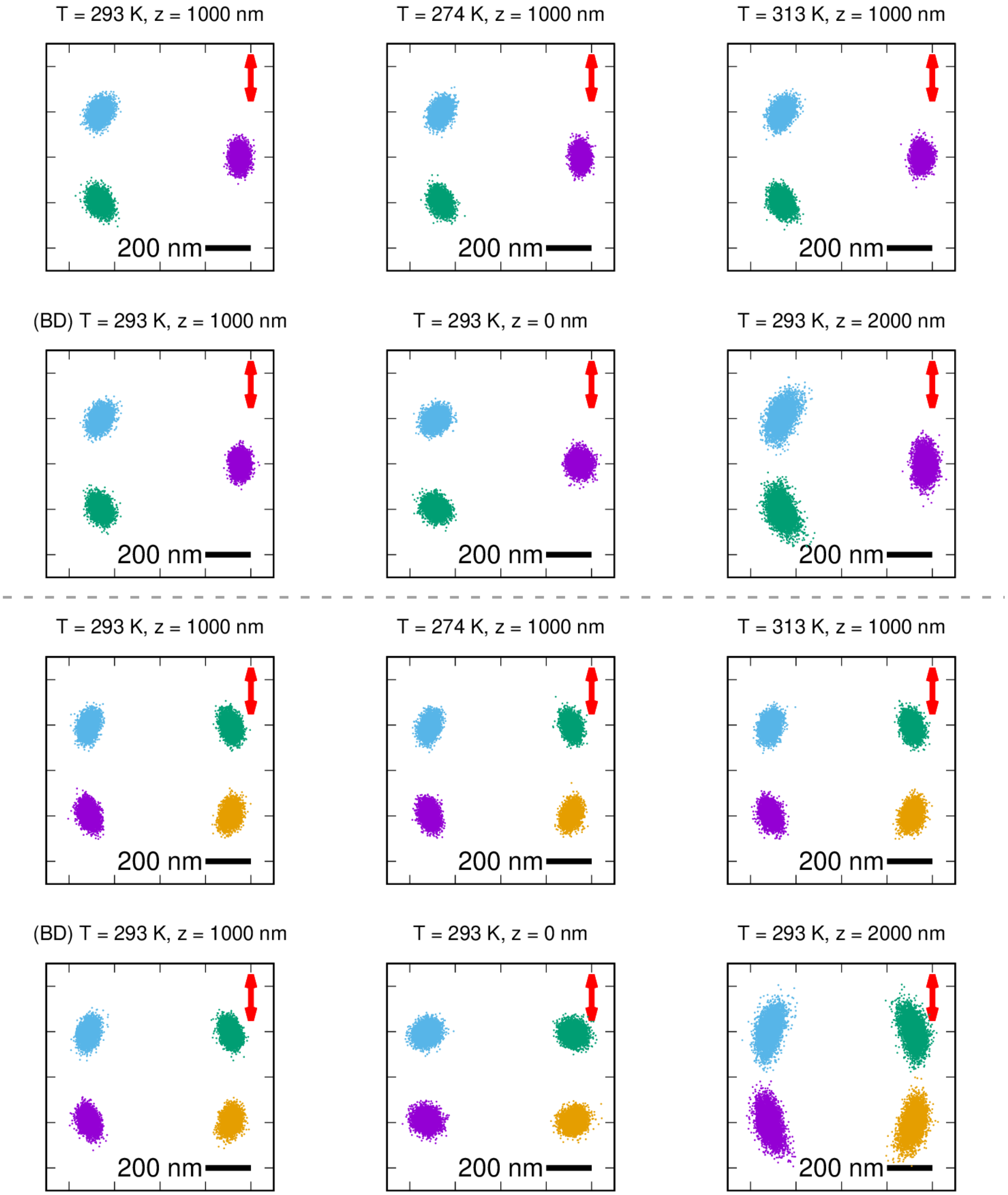

Figure S8: Simulated configurations of three and four trapped particles for different temperatures T, and focal point heights above the water-glass interface z. The configurations marked (BD) refer to pure Brownian Dynamics simulations (no hydrodynamic interactions). Each particle was assigned a different color. The focal point lies at the center of each plot. The red arrow indicates the direction of polarization and the length of the scale bar is 200 nm. In the relevant range of parameter values, changing the temperature, the height of the focal point or excluding hydrodynamic interactions does not qualitatively affect the configurations.

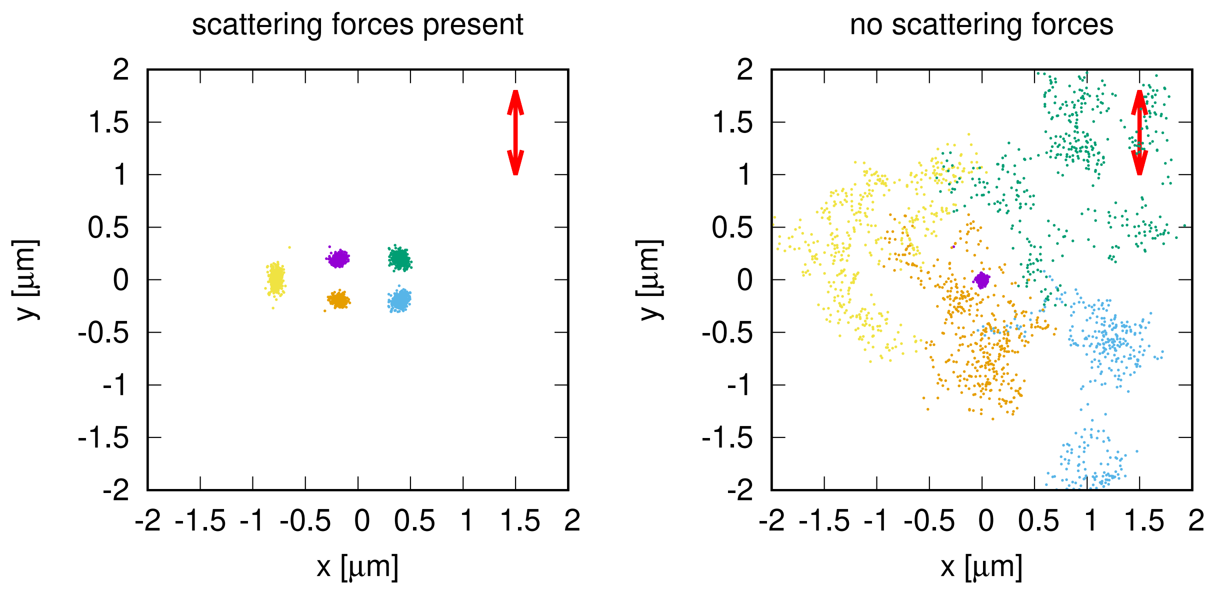

Figure S9: Positions of the centers of particles in a simulation involving five 230 nm diameter gold spheres within an optical tweezer, both with (left) and without (right) the calculation of scattering forces. The red arrow indicates the direction of polarization of the incident beam. Both simulations proceed from the same initial configuration. The addition of scattering forces lead to optical binding (*left panel*). In contrast, leaving them out leads to trapping of a single particle below the focal point.

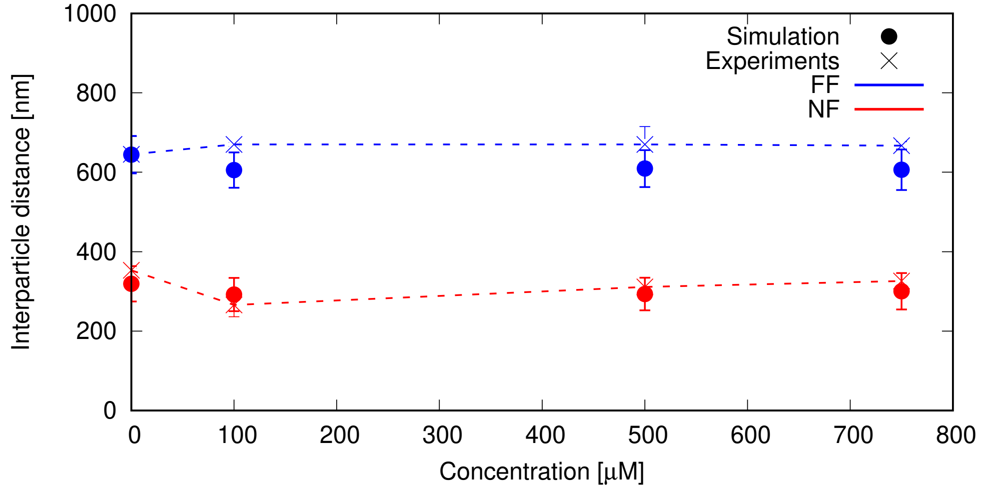

Figure S10: Center to center interparticle distance versus NaCl salt concentration in experiments and simulations for NF (*red*) and FF (*blue*) configurations.

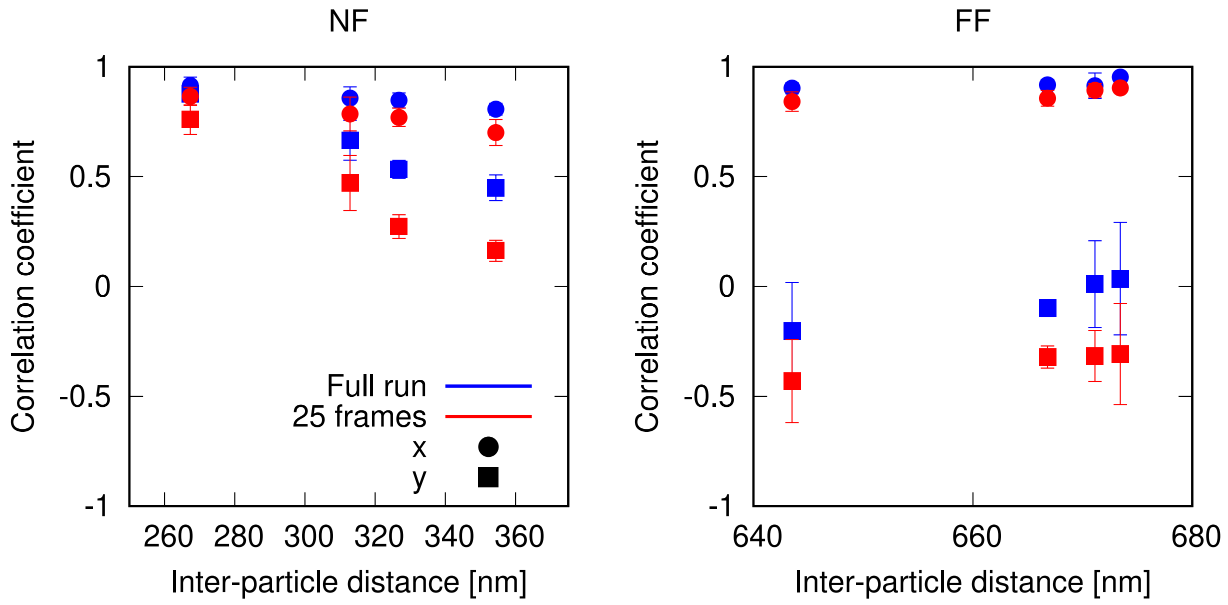

Figure S11: Experimental Pearson correlation coefficients between the x coordinates of two trapped nanoparticles, and their y coordinates, for the NF and FF configurations at different interparticle distances (due to changes in the NaCl salt concentration). Full run data was calculated for observations over 1000 frames (10 s), while 25-frame data corresponds to 0.25-second intervals. 25-frame data is more representative considering the particle movements, especially for y correlation coefficients in the FF case.

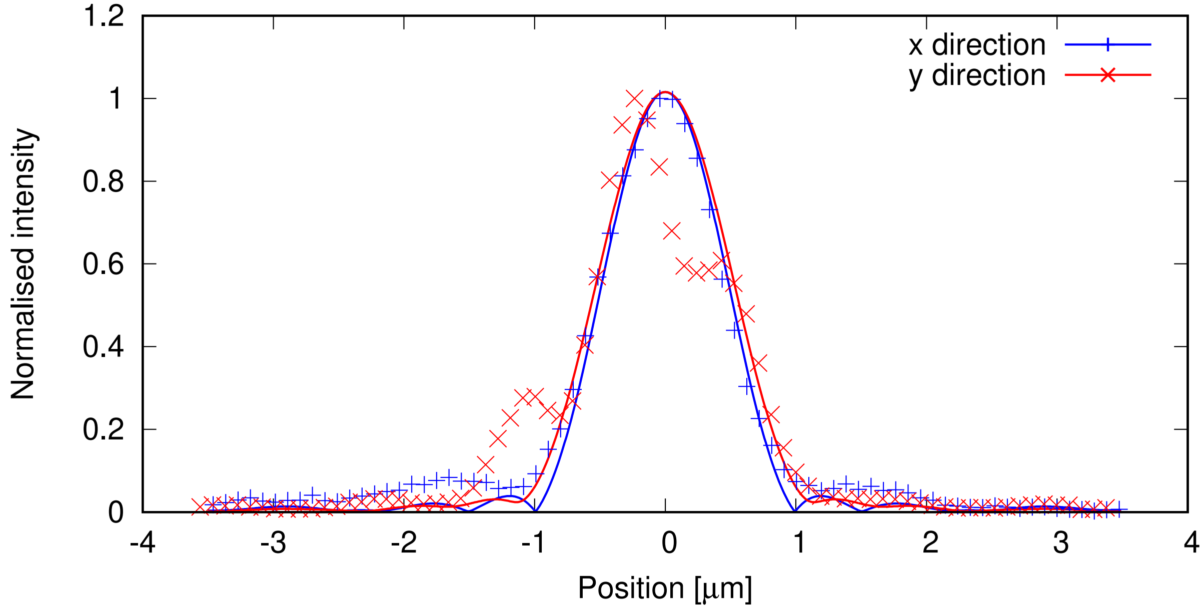

Figure S12: Normalised laser intensity profile on the focal plane along the x and y directions, with the focus at the origin. The beam was polarized in the y direction. Solid lines represent the analytical field used in simulations, and points were obtained from direct measurements in the experiment.
